# Diversity and Frequency of HLA‐DRB1*15:03‐DRB5 Haplotypes in a Large Cohort: The Case of the Absent HLA‐DRB5 Revisited

**DOI:** 10.1111/tan.70357

**Published:** 2025-08-10

**Authors:** Michael Ponisciak, Abdelhamid Liacini, Eric Pimpinella, Rehan Mujeeb Faridi, Melanie Hagerty, Carly Carozza, Noureddine Berka

**Affiliations:** ^1^ American National Red Cross, Histocompatibility Laboratory Philadelphia Pennsylvania USA; ^2^ Department of Pathology and Laboratory Medicine University of Calgary Calgary Alberta Canada

**Keywords:** DRB1*15:03 positive, DRB5 negative, linkage disequilibrium, putative haplotypes

## Abstract

The HLA class II region contains nine DRB genes: DRB1, DRB3, DRB4 and DRB5 express functional gene products, whereas DRB2, DRB6, DRB7, DRB8 and DRB9 are pseudogenes. In this study, we assessed the frequency and diversity of two functional genes, DRB1 and DRB5; in particular, HLA‐DRB1*15:03 with absence of the associated HLA‐DRB5*01:01 allele (DRB1*15:03 positive DRB5*01:01 negative). We aimed to determine the frequency of DRB1*15:03 positive DRB5*01:01 negative in this cohort and to define any putative full or partial haplotypes that may show this genotype pattern. We performed HLA typing by NGS using One Lambda AllType or CareDx AlloSeq Tx17 on 6268 solid organ transplant (SOT) samples using DNA extracted from either buccal swabs or whole blood. The absence of HLA‐DRB5 was confirmed by One Lambda LABType rSSOP. Confirmation of DRB1*15 homozygotes was performed by CareDx Copy Number (RUO) tool. In the present cohort, 554 (8.8%) individuals were identified as HLA‐DRB1*15:03 positive. Of those, 48 individuals (8.7% of HLA‐DRB1*15:03 positive) did not have the associated DRB5*01:01 allele present. We believe that this percentage could be as high as 11.2% when considering DRB1*15:XX homozygotes. Although linkage disequilibrium analysis showed strong LD between DRB1*15:03 and DRB5*01:01 (*D*′ = 0.89), supporting their co‐inheritance on the same haplotype block, a moderate correlation coefficient (*r* ^2^ = 0.302) implied that DRB5*01:01 is not always present in all DRB1*15:03 carriers. Extended haplotypes associated with this phenomenon were constructed and characterised. Certain HLA‐A, B, DRB1 haplotypes were strongly predictive of absent DRB5. To our knowledge, this is the largest cohort ascertaining the diversity and frequency of the HLA‐DRB1*15:03 apparent haplotypes. Our results show a considerable proportion of DRB1*15:03 positive individuals lack the DRB5 gene. The results of this study are useful for unrelated donor research, transplantation, anthropological and disease association studies.

## 1 Introduction

The existence of the linkage disequilibrium (LD) phenomena across the human genome is important and useful in disease association and histocompatibility studies. It has long been established that the antigens of the HLA‐DR2 serotype, encoded by HLA‐DRB1*15 and HLA‐DRB1*16, are in strong LD with serotype HLA‐DR51, encoded by the HLA‐DRB5 gene [1]. It has also been shown that HLA‐DRB1*15 alleles are associated with HLA‐DRB5*01 and HLA‐DRB1*16 alleles are associated with HLA‐DRB5*02. This includes the expected association of HLA‐DRB1*15:03 with HLA‐DRB5*01:01. What has not been extensively studied is the circumstance of HLA‐DRB1*15:03 positive specimens with absent HLA‐DRB5 [1, 2]. A significant proportion of HLA‐DRB1*15:02 and DRB1*15:03 individuals fail to express a DR51 molecule due to the presence of a null allele or the absence of a DRB5 gene [2, 3]. Given the disease association and histocompatibility importance of these genes, we aimed to investigate the NGS‐derived HLA genotypes of a large cohort of solid organ transplant (SOT) patients and donors to determine the frequency of DRB1*15:03 in this cohort, the frequency of positive DRB1*15:03 with absent HLA‐DRB5, and any full or partial putative haplotypes observed in the DRB1*15:03 positive DRB5 negative group.

## 2 Materials and Methods

Our cohort consisted of 6268 blood or buccal swab specimens of SOT patients and donors processed from October 2021 through March 2024. Specimens were extracted on either the ThermoFisher KingFisher Flex or Promega Maxwell RSC, using either the MagMax DNA Multi‐Sample Ultra 2.0 kit or the Maxwell RSC Buffy Coat DNA kit. HLA genotyping was performed by NGS using either ThermoFisher AllType or CareDx AlloSeq Tx17. Specimens were typed at 11 loci (HLA‐A, ‐B, ‐C, DRB1, DRB3/4/5, DQA1, DQB1, DPA1, DPB1). Confirmation of absent DRB5 for HLA‐DRB1*15:03 heterozygotes was established using ThermoFisher LABType SSO DRB3, 4, 5. Confirmation of single copy DRB5 for select HLA‐DRB1*15, DRB1*15:03 specimens was established by performing copy number assessment using CareDx Assign Copy Number (RUO) tool. LD calculations were performed using the dplyr package (R Foundation for Statistical Computing, Vienna, Austria). Samples were filtered to identify carriers of DRB1*15:03 using allele strings from the phased data. Co‐occurrence with DRB5*01:01 was assessed using the corresponding allele fields. LD statistics (*D*, *D*′ and *r* ^2^) were calculated based on a 2 × 2 contingency table of DRB1*15:03 and DRB5*01:01 presence. Extended haplotypes were constructed by concatenating alleles from HLA‐A, ‐B, ‐C, ‐DRB1 and ‐DRB5 loci on each chromosome. Frequencies of unique haplotypes were computed for the full cohort and stratified by DRB1*15:03 carrier status. Where individuals were heterozygous at multiple loci, phased haplotypes were inferred based on population‐specific known associations between alleles or the most likely combinations based on published haplotype frequencies. Putative haplotype frequencies were calculated by dividing the number of occurrences of each haplotype by the total number of haplotypes observed in the study cohort. This study was approved by the American Red Cross Biomedical Services Institutional Review Board, protocol #2024‐035.

## 3 Results

In our cohort, 554 out of 6268 individuals (8.8%) carried at least one copy of HLA‐DRB1*15:03. Of the 554 DRB1*15:03 positive individuals, 48 individuals (8.7%) lacked an associated DRB5*01:01 allele. LD analysis showed strong LD between DRB1*15:03 and DRB5*01:01 (*D*′ = 0.89), supporting their co‐inheritance on the same haplotype block. Conversely, a moderate correlation coefficient (*r* ^2^ = 0.302) implied that DRB5*01:01 is not always present in all DRB1*15:03 carriers, as observed in this dataset. Based on this large cohort, we were able to characterise multiple putative haplotypes associated with this phenomenon (see Table 1). Although HLA‐A locus alleles were varied, these putative haplotypes included the following predictive markers: B*49:01, C*07:01 (38.8%, 19/48); B*15:03, C*02:10 (25%, 12/48); B*53:01, C*06:02/04:01 (10.4%, 5/48); B*51:01, C*16:01 (8.3%, 4/48); B*35:01, C*04:01/C*06:02 (4.16%, 2/48); B*57:03, C*02:10/C*14:02 (4.16%, 2/48); B*07:01, C*12:03 (2.06%, 1/48); B*14:01, C*08:02 (2%, 1/48); B*44:03, C*14:03 (2%, 1/48); and B*81:01, C*18:01 (2%, 1/48). Notably, three distinct putative haplotypes emerged, haplotype 1 with HLA‐A*01:02 found in 7 of 19 individuals (36.84%) and A*02:02 in 4 of 19 (21%), haplotype 2 with A*74:11 in 9 of 12 (75%), and haplotype 3 with A*68:02 in 3 of 4 (75%) showed a higher frequency in this cohort. Previous studies have reported that haplotypes of HLA‐DRB1*15:03 without DRB5 are frequently associated with HLA‐B*49:01, and our data supports this finding. The genetic distance between HLA‐A, HLA‐DRB1 and HLA‐DPB1 makes these genes more prone to crossover events, explaining their sporadic absence in putative haplotypes.

**TABLE 1:** Putative haplotypes of HLA‐DRB1*15:03 positive DRB5 negative.

Putative HLA haplotypes	HLA‐A*	HLA B‐C association (frequencies)	HLA‐DRB1*	HLA‐DRB5*	HLA‐DQA1*	HLA‐DQB1*	Distribution (%)
Haplotype 1	01:02	B*49:01‐C*07:01 38.8% (19/48)	15:03	ABSENT	01:02	06:02	7 (36.84)
	02:02						4 (21)
	26:01						2 (10.5)
	30:02						2 (10.5)
	34:02						2 (10.5)
	02:01						1 (5.26)
	68:02						1 (5.26)
Haplotype 2	74:11	B*15:03‐C*02:10 25% (12/48)	15:03	ABSENT	01:02	06:02	9 (75)
	74:01						2 (16.66)
	36:01						1 (8.33)
	02:02	B*53:01‐C*06:02/04:01 10.4% (5/48)	15:03	ABSENT	01:02	06:02	1 (20)
	33:03						2 (40)
	34:02						2 (40)
Haplotype 3	68:02	B*51:01‐C*16:01 8.3% (4/48)	15:03	ABSENT	01:02	06:02	3 (75)
	03:01						1 (25)
	30:01	B*35:01‐C*04:01/C*06:02 4.16% (2/48)	15:03	ABSENT	01:02	06:02	1 (50)
	30:02						1 (50)
	74:01	B*57:03‐C*02:10/C*14:02 4.16% (2/48)	15:03	ABSENT	01:02	06:02	1 (50)
	23:01						1 (50)
	03:01	B*07:01‐C*12:03 2.06% (1/48)	15:03	ABSENT	01:02	06:02	1 (100)
	31:01	B*14:01‐C*08:02 2.06% (1/48)	15:03	ABSENT	01:02	06:02	1 (100)
	68:02	B*44:03‐C*14:03 2.08% (1/48)	15:03	ABSENT	01:02	06:02	1 (100)
	03:01	B*81:01‐C*18:01 2.06% (1/48)	15:03	ABSENT	01:02	06:02	1 (100)

## 4 Discussion

The occurrence of HLA‐DRB1*15:03 with absent HLA‐DRB5 should no longer be considered rare [4]. Our findings showed that among 6268 individuals, 554 (8.8%) carried at least one DRB1*15:03 allele. Of these, 506 (91.3%) also carried DRB5*01:01, whereas 48 (8.7%) did not. LD analysis revealed a high *D*′ (0.897) and moderate *r* ^2^ (0.302), indicating strong historical linkage with incomplete predictability. Subsequently, the study identifies multiple putative haplotypes associated with this phenomenon. The most frequently observed haplotypes include the combination of HLA‐B*49:01 with C*07:01 in 38.8% of cases, followed by HLA‐B*15:03 with C*02:10 in 25% and HLA‐B*51:01 with C*16:01 in 8.3%. The frequency of DRB1*15:03 positive DRB5 negative genotypes in our cohort was unexpected and could lead to a broader discussion. There is value in knowing the HLA‐DRB3/4/5 genotype of all transplant recipients and donors. It has been demonstrated that HLA‐DR51 contains highly immunogenic epitopes that can induce de novo antibody [5, 6]. It has also been observed that mismatches at HLA‐DRB3/4/5 loci can increase adverse outcomes in haematopoietic stem cell transplant (HSCT), including increased transplant‐related mortality [7, 8]. However, while HLA‐DRB3/4/5 must be typed for SOT donors, it is not required to be typed for recipients. In the context of HSCT recipients and donors, typing of HLA‐DRB3/4/5 is left to the discretion of the donor registry and/or transplant program. A standardised approach to typing HLA‐DRB3/4/5 for all transplant recipients and donors would be welcome and beneficial [9].

To our knowledge, this is the largest study that has described HLA‐DRB1*15:03 positive DRB5 negative putative haplotypes derived from HLA genotyping by NGS methods. In this report, it is important to mention some of the potential limitations of this study, which include potential population‐specific bias (as ethnicity was not available for this cohort), the limited geographic area specific to the Northeastern United States, the uncertainty in haplotype phasing in individuals who are heterozygous at multiple HLA loci, and reliance on non‐population‐specific LD assumptions.

## Author Contributions

All authors have read, critically revised and approved the final manuscript. M.P. and N.B. designed the study. M.P., A.L., R.M.F. and E.P. analysed the data. M.P. wrote the manuscript.

## Conflicts of Interest

The authors declare no conflicts of interest.

## Data Availability

The data that support the findings of this study are available on request from the corresponding author. The data are not publicly available due to privacy or ethical restrictions.
